# Cost-Utility of Intermediate Obstetric Critical Care in a Resource-Limited Setting: A Value-Based Analysis

**DOI:** 10.5334/aogh.2907

**Published:** 2020-07-20

**Authors:** Claudia Marotta, Francesco Di Gennaro, Luigi Pisani, Vincenzo Pisani, Josephine Senesie, Sarjoh Bah, Michael M. Koroma, Claudia Caracciolo, Giovanni Putoto, Fabio Amatucci, Elio Borgonovi

**Affiliations:** 1 Section of Operational Research, Doctors with Africa Cuamm, Padova, IT; 2 Section of Operational Research, IRCCS Neuromed, IT; 3 Department of Intensive Care, Amsterdam University Medical Centers, Location AMC, Amsterdam, NL; 4 Mahidol–Oxford Tropical Medicine Research Unit (MORU), Mahidol University, Bangkok, TH; 5 Princess Christian Maternity Hospital, University of Sierra Leone, Freetown, SL; 6 Department of Anesthesia and Intensive Care, University of Sierra Leone, Freetown, SL; 7 Department of Law, Economics, Management and Quantitative Methods (DEMM), University of Sannio, IT; 8 Centre for Research on Health and Social Care Management (CERGAS), Bocconi University, IT

## Abstract

**Background:**

Sierra Leone faces among the highest maternal mortality rates worldwide. Despite this burden, the role of life–saving critical care interventions in low–resource settings remains scarcely explored. A value-based approach may be used to question whether it is sustainable and useful to start and run an obstetric intermediate critical care facility in a resource–poor referral hospital. We also aimed to investigate whether patient outcomes in terms of quality of life justified the allocated resources.

**Objective:**

To explore the value-based dimension performing a cost-utility analysis with regard to the implementation and one-year operation of the HDU. The primary endopoint was the quality-adjusted life-years (QALYs) of patients admitted to the HDU, against direct and indirect costs. Secondary endpoints included key procedures or treatments performed during the HDU stay.

**Methods:**

The study was conducted from October 2, 2017 to October 1, 2018 in the obstetric high dependency unit (HDU) of Princess Christian Maternity Hospital (PCMH) in Freetown, Sierra Leone.

**Findings:**

523 patients (median age 25 years, IQR 21–30) were admitted to HDU. The total 1 year investment and operation costs for the HDU amounted to €120,082 – resulting in €230 of extra cost per admitted patient. The overall cost per QALY gained was of €10; this value is much lower than the WHO threshold defining high cost effectiveness of an intervention, i.e. three times the current Sierra Leone annual per capita GDP of €1416.

**Conclusion:**

With an additional cost per QALY of only €10.0, the implementation and one-year running of the case studied obstetric HDU can be considered a highly cost-effective frugal innovation in limited resource contexts. The evidences provided by this study allow a precise and novel insight to policy makers and clinicians useful to prioritize interventions in critical care and thus address maternal mortality in a high burden scenario.

## Background

As international commitments in the health sector become more complex in the face of increasingly constrained aid resources, funding stakeholders increased the demand for value-for-money (VfM) assessments of global health interventions [1,2,3]. This holds true also for maternal and newborns health [4,5]. Community based, antenatal care packages, and other primary care interventions to reduce maternal mortality have been demonstrated to be highly cost-effective [6]. This also applies to most hospital-based interventions, irrespective of their resource-intensiveness. In fact, with accessible and good quality clinical services, most maternal deaths may be averted—e.g., with skilled attendance to allow recognition and treatment of complications, along with a timely referral to hospitals for more complex care [6]. This hospital-based care includes various forms of obstetric intensive care support.

In a resources-limited setting, up to 15% of pregnant women suffer from some form of critical illness [7], conditions such as eclampsia, hemorrhage, coagulopathy, and sepsis, which may benefit from a more intensive setting of care. In high–income settings, this is provided by intensive care units (ICUs) [8,9]. However, ICUs require significant technological, human, and technical investments, seldom affordable in resource constraints contexts. In poorer settings, high dependency units (HDUs) may represent frugal innovations incorporating few but essential lifesaving interventions to critically-ill women [9,10]. These include a high patient to nurse ratio, close monitoring of vital signs, a personalized intravenous fluid, and vasopressor therapy management, rational use of oxygen and antibiotics, a fundamental point of care laboratory [11], adequate pain management, blood transfusions, and renal output monitoring in the early postoperative period, may impact outcomes for critical pregnant women in a referral Comprehensive Emergency Obstetric Care Service (CEmOC).

However, there are no accurate estimates of health effects and costs sustained by services providing intermediate obstetric critical care in resource-limited countries.

In particular, Sierra Leone is the country with highest maternal mortality ratio (MMR) worldwide – accounting 1.360 deaths per 100.000 live births in 2015, and healthcare system has been strongly proved by a prolonged civil war (1991–2002) followed by Ebola virus disease outbreak (2014–2016). These events have profoundly affected the already fragile healthcare system, leading to a significant worsening of maternal health indicators [11,12,13].

The case of the HDU of an urban, high-volume maternity referral hospital in Freetown, Sierra Leone, maybe paradigmatic in order to assess the cost-utility of such intervention. In this study, we hypothesized that the value of the extra cost per QALY gained from the implementation and one-year running of the HDU in a large maternity hospital would amount to less than three times the country’s average per capita gross domestic product (GDP) at purchasing power parity. This cutoff follows suggested benchmarks for adequate value for money (VfM) in global health interventions [14].

This study aimed to evaluate from a value-based perspective whether it is sustainable, economic, and ‘useful’ to introduce an obstetric intermediate critical care setting in contexts with high morbidity and mortality coupled to limited resources to face these challenges. We also questioned whether the outcomes obtained in terms of quality of life justify the investment of the expected costs.

## Methods

### Study design

We performed a retrospective cost-utility analysis for the implementation and one-year (2nd October 2017 to 2nd October 2018) operation of the HDU of a large maternity hospital in an African urban context (Princess Christian Maternity Hospital [PCMH], Freetown, Sierra Leone). The study received ethical approval and a waiver of informed consent from the Sierra Leone Ethics and Scientific Review Committee (on December 18, 2018). The study was registered on ClinicalTrials.gov (study identifier NCT04121234).

### Study Setting

With 129 beds, the PCMH is the largest maternity referral hospital in Sierra Leone, with a reference population of 1.5 million inhabitants. PCMH is a primarily obstetric institution with approximately 9,000 admissions and 6,500 deliveries per year [15,16]. One-third of the parturients develop major obstetric emergencies, including peripartum haemorrhage, sepsis, and pre-eclampsia [15,16]. Theatre and anesthetic facilities are essential. The only public ICU in Freetown with a very basic setup is located at the nearby Connaught Hospital.

### Intervention

**Figure 1:**
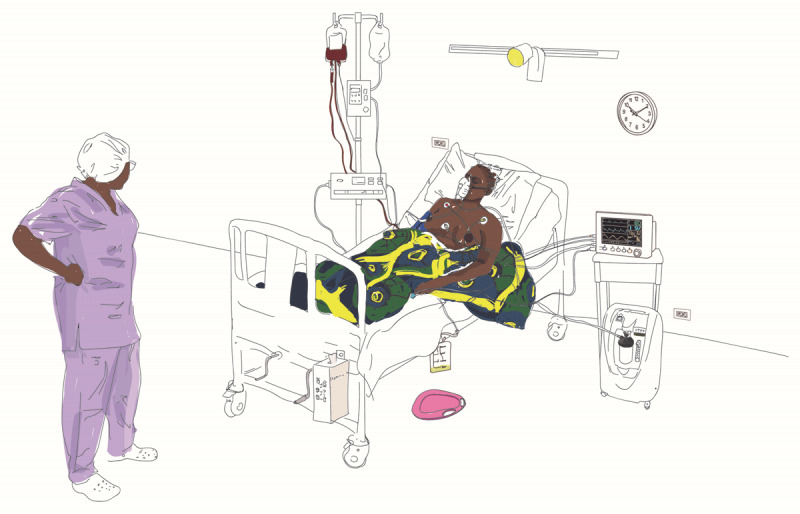
Overview of a PCMH HDU bed with essential standards of care provided (see text).

The intervention assessed was the implementation and one-year operation of a nurse-based HDU in a high-volume urban referral maternity hospital. The HDU was set up to centralize at-risk patients, or patients with established organ failures, especially after lifesaving surgery and anesthesia. The HDU aimed to ensure the maximal level of assistance possible in this context in order to reduce maternal mortality – or guarantee a dignified terminal phase in case of death. The HDU is a 4-bed medium care unit, with an additional four step–down beds, with a nurse to patient ratio of 1:2 available 24/7. Common interventions include close monitoring of vital signs and organ function, intravenous fluids, vasopressor therapy, antibiotics, rational use of oxygen, and a very basic point of care laboratory [17]. Electricity and clean water were continuously available, while oxygen was generated through bedside oxygen concentrators with a maximal output of 10 l/min and maximal purity of 96%. The basic setup of a typical HDU bed in PCMH is shown in Figure 1. No mechanical ventilators or dialysis apparatus were available in the unit or hospital at the moment this study ran. A basic neonatal ICU was available as a separate entity. Physicians performed a clinical round twice a day and were called when needed. The HDU is supported by ‘Doctors with Africa – CUAMM’ (DwA – CUAMM), an Italian non–governmental organization. A central room of the hospital was chosen and renovated with independent water and power system. Specific training was done to a selected pool of nurses with the collaboration of trainers from the Network for Intensive Care Skills Training [18].

### Study endpoints

The primary endpoint was the quality-adjusted life-years (QALYs) of patients admitted to the HDU during the study period, against direct and indirect extra costs of the HDU admission. Secondary endpoints included key treatments received during HDU stay.

### Data collection

The study included all women during pregnancy or up to 42 days after the termination of pregnancy [19], admitted to the hospital, and HDU in the one-year study time-frame. The primary data source was the HDU patient chart, with data crosschecked with the hospital patient charts and the HDU admission book for quality control purposes. Data on hospital deliveries, admissions, and mortality were taken from the hospital register and the maternal mortality hospital database. The data was collected by a dedicated researcher (CM) and included patient demographics, admission date, and source, main reasons for admission to hospital, defined as by the WHO handbook on Monitoring emergency obstetric care [19], (detailed in Supplementary Table 1); the main reason for admission to the HDU, classified as haemodynamic instability or haemorrhage; sepsis; acute renal failure; neurological impairment; respiratory distress; severe malaria; coagulopathy; other diagnoses. These were system–based diagnoses based on the clinical assessment of the attending physician rather than strict research definitions.

Specific treatments received at any point during HDU stay included: oxygen supplementation, use of vasopressors, blood transfusions, antibiotic therapy, eclamptic seizures prevention with intravenous or intramuscular magnesium sulphate and anti–hypertensive treatment with intravenous hydralazine. Time from hospital admission to HDU admission was calculated. Length of stay (LOS) and patient outcomes at discharge (classified as a death in HDU, discharge to ward, or transfer to other facilities).

### Assessment of Value

Among the variety of methods to assess value, we used QALY [20]. QALYs are a composite measure of health outcomes, which combine the length of time spent in a health state with the quality of life experienced in that health state. Specifically, the estimation of QALYs multiplies two variables, namely:

- *Years of life gained due to the health intervention*: calculated as the difference between the age of the woman when the critical health event occurred and the life expectancy in Sierra Leone at that time (53.8 years) [21,22,23].
- *Health-related quality-of-life weights*: health-related quality-of-life weights associated with each health state in the model – on a cardinal scale of 0–1, where 0 indicates death and 1 indicates full health – were derived from the peer-reviewed literature (Table 1), and age-specific baseline quality-of-life estimates for reproductive health.

**Table 1:** Health-related quality-of-life weights.

Quality of Life weights	Health-related reasons

**0.0**	Deaths
**0.30**	Referral to Intensive Care Unit
**0.40**	Hysterectomy in patients <30 years
**0.80**	B-Lynch surgical procedure in patients <30 years
**0.90**	Uterine ruptures in patients <30 years
**0.90**	Sepsis (29)
**0.95**	Pre-eclampsia/eclampsia (30)
**0.50**	Other severe diagnosis (disseminated intravascular coagulation, emiparesis)
**1.0**	Full recovery at discharge

### Costs

Direct and indirect costs were included and classified as implementation cost (drugs, equipment, medical materials and consumable, human resources, renovation, training, and other – e.g., this included an electricity generator) and running costs (drugs, medical materials and consumable, human resources, maintenance, training). Custom import taxes were also included. No adjusting of unit costs for inflation was performed.

We analysed only *extra expenditure for HDU implementation and one-year running*, calculated as the amount of direct and indirect cost provided by the NGO in addition to the Free Health Care Initiative provided by the National Health System and to the hospital infrastructures already available. Thus by using the term ‘*cost*,’ we refer to the *extra cost* in addition to the current funding. Neither hospital budget nor specific government expenditure for HDU information was available – even upon request – and for this reason, the total expenditure for HDU could not be estimated.

We undertook a retrospective costing review using as primary data source the DwA-UAMM project budget and accountability, including invoices. As for the pharmaceutical expenditure, the DwA-CUAMM pharmacy register was used, and data were crosschecked with the HDU pharmacy request book. The financing spectrum for the first year was mixed: DwA–CUAMM mainly financed renovation, training, and material procurement, while the nurse pool and drugs were financed both by hospital budget and DwA–CUAMM. A pool of 13 nurses was trained and dedicated to HDU and received an allowance from DwA–CUAMM for the extra working hours in addition to their former salary. Similarly, drugs from the hospital pharmacy were assigned to the HDU as for the other hospital wards, and extra drugs provision was supplied from DwA–CUAMM.

### Cost-Utility Analysis

Cost per QALY was calculated by dividing the total costs of the intervention (investment and operations) by the number of patients treated in the study time window. The cost per QALY was also analyzed according to the main admission diagnosis. In order to provide an estimation of running costs, the cost per QALY was also calculated, excluding the investment costs.

The cost-utility of the intervention was evaluated from a Value for Money (VfM) point of view, verifying if each QALY gained had an extra cost less than three times the country’s average per capita gross domestic product (GDP) at purchasing power parity, as suggested from WHO in global health interventions [14]. The Sierra Leone GDP in 2018 was $4 billion [20], with a GDP per capita of 523 US dollars ($) or 472 euros. This ranks Sierra Leone 187^th^ among 196 censed countries [21]. Thus an intervention yielding a QALY for <$523 is considered *very cost-effective*. Interventions yielding a QALY at a cost greater than three times GDP per capita (>$1569) are considered *not cost-effective*, while those falling between $523 and $1569 are considered *cost-effective*.

## Results

### Patients Characteristics and Clinical Outcomes

From the 2^nd^ October 2017 to 30^th^ September 2018, 523 patients (median age 25 years, IQR 21-30) were admitted, an average of 44 patients a month. Patients’ clinical profile and specific fatality rates are objects of a separate analysis. Fifty-five patients died in HDU (10.5%). Four out of five patients (n = 428, 81.9%) improved and were transferred to the ward after a median stay of two days (IQR 1-3). Thirty-three patients (6.3%) were transferred to an external ICU or to other hospitals after a median stay of two days (IQR 1-4), and seven patients (1.3%) were discharged directly at home after a median stay of five days (IQR 4-6). Key procedures and treatments administered in both dead and alive cases are reported in Table 2.

**Table 2:** Use of key procedures and treatments provided in the HDU compared between survivors and non-survivors.

Treatment	All patients (n = 523)	Alive cases (n = 468)	Dead cases (n = 55)

**Oxygen**	116 (22.2%)	84 (72.4%)	32 (27.6%)
**Vasopressors**	68 (13.0%)	45 (66.2%)	23 (33.8%)
**Transfusions**	263 (50.3%)	241 (91.6%)	22 (8.4%)
**Antibiotics**	109 (20.8%)	103 (94.5%)	6 (5.5%)
**Magnesium Sulphate protocol**	72 (13.8%)	63 (87.5%)	9 (12.5%)
**Hydralazine protocol**	74 (14.1%)	68 (91.9%)	6 (8.1%)

### Costs

Values for investment and one-year running costs are detailed in Table 3. The total cost summing investment with operation costs was of €120,082. Total investment costs accounted for approximately half of the total costs, with one quarter spent on equipment, one quarter for renovation work, 14% for the electricity generator, 13% for the nurse training, and only 11% for medical materials and drugs. Instead, most of the one-year running costs were explained by medical materials and consumables, followed by maintenance, human resources, and training. Detailed costs per each category are available in Supplementary Table 2, while the drugs available are listed in Supplementary Table 3.

**Table 3:** Values for investment and one-year running costs of the HDU in the study.

	Value in €	%

**INVESTMENT COSTS**	**64.064,65**	**100**

**Drugs, medical materials and consumable**	6.763,50	11
**Equipment**	16.355,31	26
**Human resources**	7.644,44	12
**Other – extra generator**	9.182,12	14
**Renovation work**	15.971,35	25
**Training**	8.147,92	13
**ONE-YEAR RUNNING COSTS**	**56.017,28**	**100**

**Equipment, medical materials, and drugs**	33.956,54	61
**Human resources**	5.094,95	9
**Maintenance**	13.182,83	24
**Training**	3.782,95	7
**TOTAL COSTS**	**120.081,93**	

### Values of QALY

The estimation of the years of life gained with the intervention was 28.8 years (median, range 8.8–39.8). The total of years gained was 14,160.6 years resulting in a cost for a year of life gained value of €8.4.

The value of QALY gained on the overall sample was 22.8. The mean values of QALY gained for each of the main admission diagnosis are reported in Table 4. The category “others” – disseminated intravascular coagulation (DIC), sickle cell disease, severe malaria – had the highest cost per QALY of €12.5, followed by puerperal sepsis (€10.9) and PPH (€10.6); on the contrary, complications of abortion had the lowest cost per QALY value (€8.8).

**Table 4:** Values of QALY and cost per QALY per main admission diagnosis in the HDU.

Main Admission Diagnosis	n. patients n (%)	QALY (mean)	Cost per QALY (€)

**Ante-Partum Haemorrhage (APH)**	85 (16.3)	23.4	9.8
**Post-Partum Haemorrhage (PPH)**	66 (12.6)	21.7	10.6
**Pre-Eclampsia (PE)/eclampsia**	117 (22.4)	23.6	9.7
**Complications of abortion**	12 (2.3)	26.2	8.8
**Ectopic Pregnancy**	53 (10.1)	25.5	9.0
**Obstructed labour**	28 (5.4)	25.2	9,1
**Puerperal Sepsis**	49 (9.4)	21.0	10.9
**Uterine Rupture (UR)**	55 (10.5)	24.3	9.4
**Others**	58 (11.1)	18.3	12.5
**Overall**	**523**	**22.9**	**10.0**

### Cost-Utility Analysis

**Figure 2:**
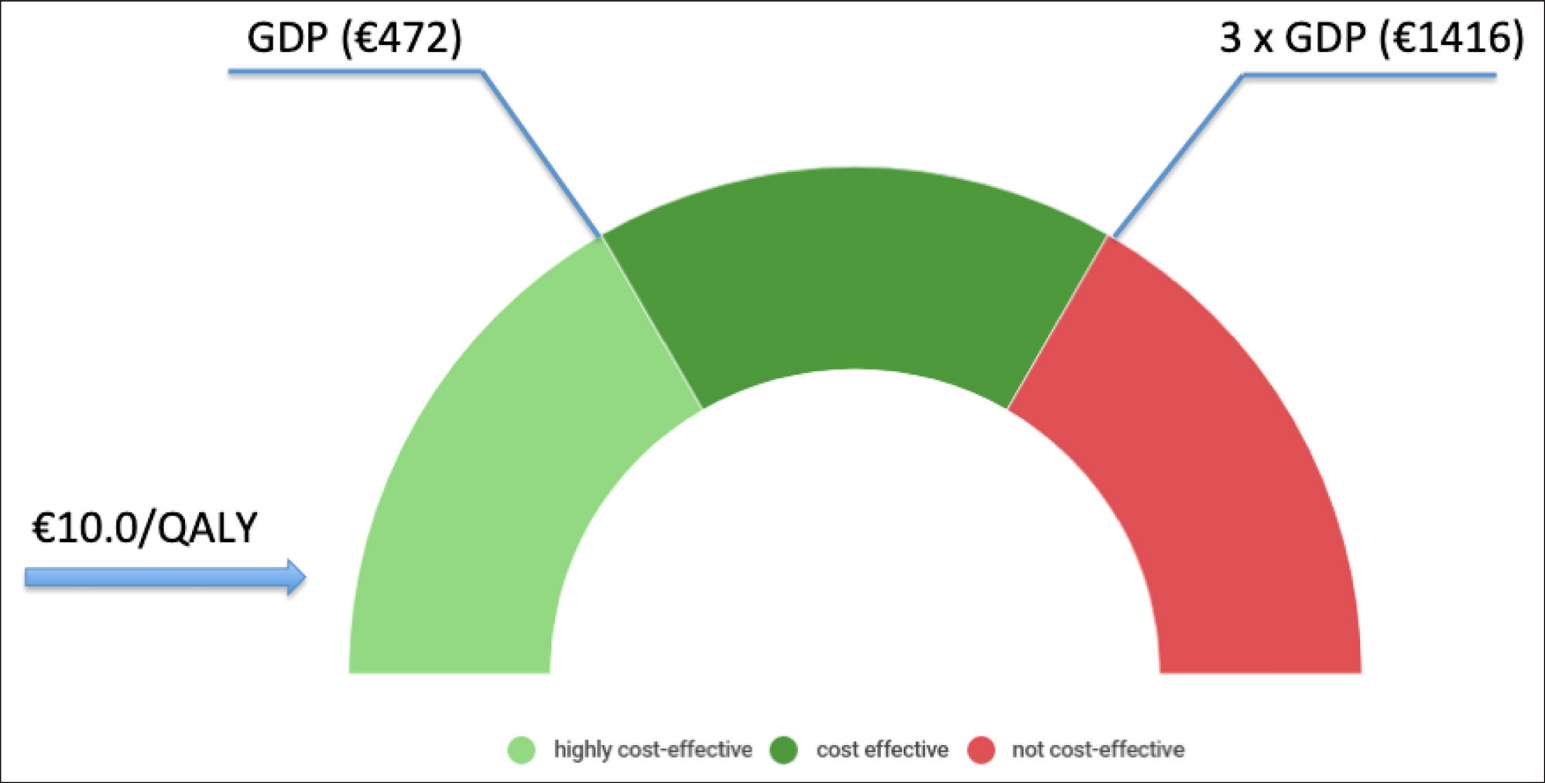
Cost for QALY of the implementation and one-year running of HDU within the framework of the World Health Organization interpretation of the cost-effectiveness of health care interventions. *If the value of cost per QALY is less than the Country’s GDP per capita, then the intervention is considered very cost-effective. If the value of cost per QALY falls between one and three times GDP per capita, then the intervention is cost-effective, and if the cost per QALY is more than three times GDP per capita, the intervention is considered not cost-effective* [12].

Dividing the total costs by the total number of patients admitted, the extra cost per admitted patient was €230, equalling a cost per QALY of €10.0. This resulted in being much lower than both thresholds defining ‘cost-effective’ and ‘very cost-effective’ interventions for Sierra Leone (Figure 2). Considering only the running costs, the cost per admission/patient was of €107, equalling a running cost per QALY of €4.7.

## Discussion

This study analyzed the sustainability of an obstetric HDU in a resource-limited setting. The cost-utility analysis yielded the following value-based findings: (1) the total cost for starting and running an HDU for more than 500 patients was of €120,082 – resulting from 53% of investment costs and 47% of one-year running costs; (2) the extra cost per admission was of €230, with an overall cost per QALY of €10; (3) the intervention can be defined as *highly cost-effective*, as the value of cost per QALY gained resulted in being much lower than the Sierra Leone annual per capita GDP.

To the best of our knowledge, no other similar research experiences have been reported in the literature. So, the strength of this analysis lies in being the first value-based evaluation of an obstetric HDU from limited resources setting with extreme maternal mortality, even if direct comparison with analogue experiences and benchmarking could not be performed. Real-world data is instrumental in informing decision-makers for resource allocation processes while defining fields of action where the greatest health gains can be achieved. In this way, it is directly related to Universal Health Coverage, since shifting from a less to a more cost-effective set of health, activities is equivalent to raising new finance.

Just above a hundred thousand euros were sufficient to start and run an HDU serving more than 500 patients. The investment cost amounted to half of this figure required for renovation and basic equipment. Most of the one-year running costs were explained by medical materials and consumables and by maintenance cost. This total figure is not excessive if we consider that ICU admission costs for advanced therapies in high–income countries may reach 80 thousand euros for a single patient [24].

The total extra cost per admission/patient in our study was €230, and this value drops to roughly €100 when accounting only for the running costs. Being intermediate – and not full ICU care, this compares favorably to the total cost per ICU admission day in India of around the US $ 200 found in one of the rare costing analyses of an ICU from a low or middle-income country [25,26]. In high-income countries, the median *daily* cost of a non-ventilated patient was recently estimated in German ICUs to euro 999 [26], thus, twenty times higher the running cost per day found in this cohort (considering a median HDU stay of two days). We were unable to assess the share of the HDU costs in the overall hospital stay costs since we lacked a precise estimation of out of HDU hospital costs. However, it is known that critical care absorbs the highest quota of hospital budgets [26,27]. This holds true also in low and middle-income countries despite these have lower ICU costs than high–income countries and thus more conservative cost-effectiveness ratios [28,29,30].

The cost per QALY gained was extremely low, i.e., €10.0 were spent for every year of life gained in perfect health. This value is very low also for a limited resource setting [14], and is surely facilitated by the young age of the patients [26] and high reversibility of obstetric conditions [8]. QALYs are a measure of the state of health of a person or group in which the benefits, in terms of length of life, are adjusted to reflect the quality of life. Being much lower than the WHO threshold of one time, the annual per capita GDP – the extra effort spent to implement and run the HDU can be considered highly cost-effective [14]. Among the variety of methods to assess value, we decided to use QALY since the study was based on the evaluation of a single intervention without direct comparison, and also because this is recommended for intensive care settings [20,31,32]. In our sample, the HDU intervention allowed an estimated average gain of 22.8 years of life in perfect health for the woman who had benefited from it. The number of life-years gained per patient is similar to the one found in an economic evaluation of a low-resource ICU in Sarajevo, where however the cost of treatment per QALY saved was higher and varied between 100 and 2514 US $ [29].

This analysis also offers a reflection in a donor ‘exit strategy’ perspective to ensure that the benefits of the intervention are not lost once the external support finishes. After the first year, the investment costs are zeroed. Hence considering only the running costs and maintaining the same volume of activity and the same patient case-mix of the year in the study, the cost per admission/patient would be only just above €100, with a cost per QALY below $5. Also, according to our results and the high morality ratio in Sierra Leone, it could be suggested to set up a national needs assessment of obstetric HDU beds and their appropriate distribution between the government district hospital of the country [31,32,33].

Our study has some notable limitations. We analysed only extra expenditure for HDU implementation and one-year running, calculated as the amount of direct and indirect cost provided from the external partner in addition to the Free Health Care Initiative provided by the NHS and to the hospital infrastructures already available. For example, surgical procedures and related costs were not included in the analysis as these are provided independently of the HDU service and are part of the routine treatment of several obstetric critical illnesses. However, it is obvious that effective supportive critical care needs fast surgical etiological treatment. Secondly, the generalizability of the findings is limited to similar settings, being this monocenter study from a single African urban setting. Finally, the retrospective nature made it impossible to examine health-related quality of life (HRQoL) after the HDU discharge, and its comparison to age-appropriate reference values from the general Sierra Leonean female population. Integrating such indicators in future investigations could allow a comprehensive value-based evaluation including also the personal value. In conclusion, our data contribute to tackling the scarcity of costing analyses regarding obstetric critical care in limited-resource settings. The obstetric HDU under study resulted in being a low-cost and highly cost-effective intervention. These findings allow a precise insight to policymakers, donors, and hospital managers that wish to consider critical care frugal interventions to address maternal mortality in low–income settings.

## Additional Files

The additional files for this article can be found as follows:

10.5334/aogh.2907.s1 Supplementary Table 1. Operational definitions of major direct obstetric complications according the WHO Handbook Monitoring emergency obstetric care.

10.5334/aogh.2907.s2 Supplementary Table 2. Detailed investment and one-year running costs.

10.5334/aogh.2907.s3 Supplementary Table 3. List of drugs used in HDU during the first year of operations.
