# Supplementary material for: Cost-Utility of Intermediate Obstetric Critical Care in a Resource-Limited Setting: A Value-Based Analysis
Source: Ann Glob Health. 2020 Jul 20;86(1):82. doi: 10.5334/aogh.2907 (PMC7380057; doi:10.5334/aogh.2907)
Supplement: Supplementary Table 1. — Operational definitions of major direct obstetric complications according the WHO Handbook Monitoring emergency obstetric care. [file agh-86-1-2907-s1.pdf]

Supplementary material to

Cost-utility of Obstetric Critical Care in a resource–limited setting: a value–based analysis

Supplementary Table 1. Operational definitions of major direct obstetric complications according the WHO Handbook Monitoring emergency obstetric care.

Major Direct Obstetric Complications definitions
**Haemorrhage**
Antepartum<br>• severe bleeding before and during labour: placenta praevia, placental abruption<br>*Postpartum* (any of the following)<br>• bleeding that requires treatment (e.g. provision of intravenous fluids, uterotonic drugs or blood)<br>• retained placenta<br>• severe bleeding from lacerations (vaginal or cervical)<br>• vaginal bleeding in excess of 500 ml after childbirth<br>• more than one pad soaked in blood in 5 minutes
**Prolonged or obstructed labour** (dystocia, abnormal labour) (any of the following)
• prolonged established first stage of labour (> 12 h)<br>• prolonged second stage of labour (> 1 h)<br>• cephalo-pelvic disproportion, including scarred uterus<br>• malpresentation: transverse, brow or face presentation
**Puerperal sepsis**
• A temperature of 38 °C or higher more than 24 h after delivery (with at least two readings, as labour alone can cause some fever) and any one of the following signs and symptoms: lower abdominal pain, purulent, offensive vaginal discharge (lochia), tender uterus, uterus not well contracted, history of heavy vaginal bleeding. (Rule out malaria)
**Complications of abortion** (spontaneous or induced)
• haemorrhage due to abortion which requires resuscitation with intravenous fluids, blood transfusion or uterotonics<br>• sepsis due to abortion (including perforation and pelvic abscess)
**Severe pre-eclampsia and eclampsia**
• Severe pre-eclampsia: Diastolic blood pressure ≥ 110 mm Hg or proteinuria ≥ 3 after 20 weeks' gestation. Various signs and symptoms: headache, hyperflexia, blurred vision, oliguria, epigastric pain, pulmonary oedema<br>• Eclampsia<br>• Convulsions; diastolic blood pressure ≥ 90 mm Hg after 20 weeks' gestation or proteinuria ≥ 2. Signs and symptoms of severe pre-eclampsia may be present
**Ectopic pregnancy**
• Internal bleeding from a pregnancy outside the uterus; lower abdominal pain and shock possible from internal bleeding; delayed menses or positive pregnancy test
**Ruptured uterus**
• Uterine rupture with a history of prolonged or obstructed labour when uterine contractions suddenly stopped. Painful abdomen (pain may decrease after rupture of uterus). Patient may be in shock from internal or vaginal bleeding
