# Supplementary material for: Cost-Utility of Intermediate Obstetric Critical Care in a Resource-Limited Setting: A Value-Based Analysis
Source: Ann Glob Health. 2020 Jul 20;86(1):82. doi: 10.5334/aogh.2907 (PMC7380057; doi:10.5334/aogh.2907)
Supplement: Supplementary Table 2. — Detailed investment and one-year running costs. [file agh-86-1-2907-s2.pdf]

Supplementary Table 2. Detailed investment and one-year running costs.

**INVESTMENT COSTS**		**€ 64.064,65**
**DRUGS**		**€ 1.131,65**
	DRUGS	€ 677,00
	TAX	€ 454,65
**EQUIPMENT**		**€ 16.355,31**
**HUMAN RESOURCES**		**€ 7.644,44**
	INTERNATIONAL NURSING COORDINATOR	€ 6.573,01
	STAFF ALLOWANCES	€ 1.071,43
**MEDICAL MATERIALS AND CONSUMABLE**		**€ 5.631,85**
	MEDICAL MATERIALS AND CONSUMABLE	€ 5.177,20
	TAX	€ 2.684,23
**OTHER - EXTRA GENERATOR**		**€ 9.182,12**
**RENNOVATION WORK**		**€ 15.971,35**
**TRAINING**		**€ 8.147,92**
	MATERIALS AND STATIONERY	€ 1.737,99
	TRAINER TRAVERL COST	€ 1.883,27
	TRAINING ALLOWANCES	€ 4.526,66
**ONE-YEAR RUNNING COSTS**		**€ 56.017,28**
**HUMAN RESOURCES**		**€ 5.094,95**
	STAFF ALLOWANCES	€ 3.987,93
	TRAINING	€ 1.107,03
**MANTENANCE**		**€ 13.182,83**
	FUEL	€ 6.675,65
	MATERIALS AND STATIONERY	€ 6.507,18
**TRAINING**		**€ 3.782,95**
	MATERIALS AND STATIONERY	€ 2.018,04
	TRAINERS TRAVERL COST	€ 1.764,91
**EQUIPMENT, MEDICAL MATERIALS AND DRUGS**		**€ 33.956,54**
	DRUGS	€ 17.080,40
	MEDICAL MATERIALS AND CONSUMABLE	€ 5.113,42
	TAX	€ 11.762,72
