# Supplementary material for: Cost-Utility of Intermediate Obstetric Critical Care in a Resource-Limited Setting: A Value-Based Analysis
Source: Ann Glob Health. 2020 Jul 20;86(1):82. doi: 10.5334/aogh.2907 (PMC7380057; doi:10.5334/aogh.2907)
Supplement: Supplementary Table 3. — List of drugs used in HDU during the first year of operations. [file agh-86-1-2907-s3.pdf]

Supplementary Table 3. List of drugs used in HDU during the first year of operations.

**Available oral and intravenous drugs used in the HDU**		
Amoxicillin 500mg, /250mg tabs	Ketamine 50mg/ml, 10ml inj 25 vials	Ferrous sulfate 200mg + Folic acid 0,4mg,
Ampicillin 1g powder for injection, 50 vials	Magnesium sulphate 500 mg/ml (50 %), 10 ml	Furosemide 20mg/2ml inj
Artemether 80mg/ml, 1ml inj, amp	Mannitol 20% solution 500ml	Gentamicin 80mg/2ml inj 10x10 amp
Artesunate 60mg pwd for inj + 2 solvents, 1 vial	Meropenem, vials 1 gr	Hidralazine, 20 mg, 2ml
Bisacodyl 5mg, enteric coated tab	Methyldopa 250 mg oral use prolonged released tablet (Blister)	Hydrocortisone 100mg (as sodium succinate) inj
Bupivacaine HCl 0,5% inj spinal heavy 4ml, 100 amp	Metyl ergometrine maleate, 0,2 mg/ml, 1ml	Hyoscine butylbromide 20mg/ml, 1ml inj
Ceftriaxone 1g powder for injection, vial	Metoclopramide HCl 10mg/2ml inj	Ferrous sulfate 200mg + Folic acid 0,4mg,
Chloramphenicol 1 g (as sodium succinate) inj, vial	Metronidazole 250 mg Tabs	Furosemide 20mg/2ml inj
ChlorpromazineHCl 50mg/2ml inj	Metronidazole 5OOmg/100mg infusion	Gentamicin 80mg/2ml inj 10x10 amp
Ciprofloxacin 2mg/ml, 100ml infusion	Misoprostol 200 microgram tab	Hidralazine, 20 mg, 2ml
Cloxacillin 500mg powder for inj	Nifedipine 20mg tab extended release	Hydrocortisone 100mg (as sodium succinate) inj
Dexamethasone sodium phosphate 5mg/ml 1ml inj,	Noradrenaline, 2mg/1ml inj	Hyoscine butylbromide 20mg/ml, 1ml inj
Dextrose 5%, bottle 500ml	Normal Saline 0,9% bottle 500 mg	Ringer Lactate 500 ml drip bottle
Diazepam, 10mg/2ml, ampoules	Omeprazole 20mg cap	Sulfadoxine 500mg / pyrimethamine 25mg tab
Diclofenac sodium 75mg/3ml inj	Oxitocine, 10 IU/ml 1ml, ampoules	Thiopental, 1gr, vial 20 ml
Digoxin 0,5mg/2ml inj	Paracetamol 500mg oral use prolonged-release tablet	Tramadol HCl 100mg/2ml inj
Dopamine HCl 200mg/5ml inj	Pethidine, hydrocloryde 50mg/ml, 2ml	Tranexamic Acid, 500mg/5ml
Doxycycline 100mg (as hyclate)	Promethazine HCl 50mg/2ml inj	Vancomicine, inj, 500 mg
Epinephrine (adrenaline) 1mg/ml 1ml inj, 10 amp	Quinine di-HCl 600mg/2ml inj 10x10 amp	
